# Emotion and liking: how director emotional expression and knowledge of (dis)liking may impact adults’ ability to follow the instructions of an ignorant speaker

**DOI:** 10.1007/s00426-020-01441-x

**Published:** 2020-11-19

**Authors:** Rebecca L. Monk, Lauren Colbert, Gemma Darker, Jade Cowling, Bethany Jones, Adam W. Qureshi

**Affiliations:** Psychology Department, Edge Hill University, Ormskirk, L39 4PY UK

## Abstract

**Background:**

Theory of mind (ToM), the ability to understand that others have different knowledge and beliefs to ourselves, has been the subject of extensive research which suggests that we are not always efficient at taking another’s perspective, known as visual perspective taking (VPT). This has been studied extensively and a growing literature has explored the individual-level factors that may affect perspective taking (e.g. empathy and group membership). However, while emotion and (dis)liking are key aspects within everyday social interaction, research has not hitherto explored how these factors may impact ToM.

**Method:**

A total of 164 participants took part in a modified director task (31 males (19%), *M* age = 20.65, SD age = 5.34), exploring how correct object selection may be impacted by another’s emotion (director facial emotion; neutral × happy × sad) and knowledge of their (dis)likes (i.e. director likes specific objects).

**Result:**

When the director liked the target object or disliked the competitor object, accuracy rates were increased relative to when he disliked the target object or liked the competitor object. When the emotion shown by the director was incongruent with their stated (dis)liking of an object (e.g. happy when he disliked an object), accuracy rates were also increased. None of these effects were significant in the analysis of response time. These findings suggest that knowledge of liking may impact ToM use, as can emotional incongruency, perhaps by increasing the saliency of perspective differences between participant and director.

**Conclusion:**

As well as contributing further to our understanding of real-life social interactions, these findings may have implications for ToM research, where it appears that more consideration of the target/director’s characteristics may be prudent.

**Electronic supplementary material:**

The online version of this article (10.1007/s00426-020-01441-x) contains supplementary material, which is available to authorized users.

## Introduction

Theory of mind (ToM) has been widely researched and is defined as our ability to understand that other agents have different mental states, knowledge, desires and intentions to ourselves (Premack & Woodruff, 1978). Present in children as young as 4 years of age (Wellman, Cross & Watson, 2001, and perhaps implicitly earlier (Onishi & Baillargeon, 2005; Surian, Caldi & Sperber, 2007), research on adults suggests that the capacity to take another’s visual perspective—a facet of ToM termed Visual Perspective Taking (VPT)—is related with executive control (e.g. Qureshi, Apperly & Samson 2010, and Qureshi & Monk 2018). Specifically, most ToM tasks create a difference between the participant’s perspective (self) and that of another agent (other). Resolving this self-other interference is, therefore, suggested to be the key to being able to respond correctly in ToM and VPT tasks.

Research in this area has, therefore, focussed a variety of participant-level variables which appear to impact the successful resolution of the self and other perspective. These include the induced emotion of the participant (Bukowski and Samson, 2016), group membership (Simpson & Todd, 2017), empathy, imitation (de Guzman, Bird, Banissy & Catmur, 2016) and processing time (Reed & McGoldrick, 2007). In an effort to understand how ToM may operate in the real world, however, the nature of the stimuli employed in standard cognitive tasks has been fairly neutral. As such, while there is a growing awareness of how the perspective taker’s ability/state may impact ToM, it is less clear how the attributes of the target (i.e. the characteristics of the person whose perspective a participant aims to adopt) may impact this capacity (though see Simpson & Todd, 2017). The current paper aims to examine whether a lack of task realism may have limited our understanding of ToM, taking a first step towards examining how manipulating displayed emotions and supplying information regarding another’s likes/dislikes may impact VPT.

In an online communication game named the Director task, participants are required to follow the instructions of an ignorant director with an opposite viewpoint, moving mutually visible objects (target objects) while ignoring objects that are only visible to themselves (competitor objects; Apperly et al., 2010). The task requires participants to resolve interference between their own (self) and the director’s (other) perspectives. However, adults consistently exhibit a failure to use ToM in this task, moving objects that the director has no knowledge of rather than the mutually visible target object (Keysar, Lin & Barr, 2003). This is referred to as an egocentric error, as responses are based on individuals’ own perspectives, rather than taking the director’s perspective (Apperly et al., 2010). The same set of studies also showed that participants were efficient at switching perspectives, suggesting that rather than reflecting an inability to take the directors perspective (e.g. impairments or deficits in ToM), errors were caused by a failure to integrate the director’s perspective with the given instructions (to constrain reference; Barr, 2008). Research in this domain, therefore, raises interesting questions about the nature of day-to-day interactions where adults’ abilities to take another’s point of view without bias from their own perspective are tested. However, such computer-based tasks do not provide any further information about the director (the things they like/dislike, for example), nor does he have any emotional expression. As both liking and emotion interpretation are key to everyday social communication and require ToM (e.g. Nguyen & Frye, 2001); it is, therefore, unclear how previous research findings may apply to real-world interactions.

Inhibitory control, the ability to stop a dominant response, has been linked to performance the Director task (Qureshi, Monk, Samson & Apperly, 2019; Symeonidou, Dumontheil, Chow & Breheny, 2016). Its role may be to allow participants to inhibit their salient self-perspective to take the belief and knowledge of the director into account. Inhibition has also been linked with other ToM tasks such as traditional false belief tasks (e.g. Carlson, Moses & Claxton 2004). While these measure belief reasoning, which can be mastered from the age of four (Wellman, Cross & Watson, 2001; though see Onishi & Baillargeon, 2005; Surian, Caldi & Sperber, 2007), the task has also been modified to assess belief-desire reasoning, where participants are told that the character has a desire to avoid an object (Leslie, German & Polizzi, 2005). Here, task difficulty is increased as participants must inhibit the self-perspective as well as predict actions based on the desire to avoid, which requires further inhibitory control (Leslie et al., 2005). However, there has been no research that has provided such desire information to participants taking part in the Director task and, as such, we aim to explore whether VPT is affected by having knowledge of another’s desires—which we define as knowing if the director likes/dislikes an object. We also postulate that while knowledge of liking requires further inhibitory control (over and above self-perspective inhibition), there may be greater difficulty in responding to an instruction that is in conflict with the information presented (e.g. when the director likes competitor or dislikes target), caused by the increased cognitive demand, relative to when the liking is not in conflict with the instruction (e.g. like target, dislike competitor). The current study, therefore, aims to examine further how perspective taking may work (or fail) when incorporating knowledge of another person’s likes or dislikes.

As well as the knowledge of the director’s likes/dislikes affecting performance, there is a growing body of research which suggests that emotion may be important in perspective taking. To wit, it should also be noted that in the computerised version of the Director task (Apperly et al., 2010), the emotional expression of the director is neutral in all conditions. Nevertheless, it is well established that emotional expressions can affect a viewer’s behaviour in various social settings (Van Kleef, 2009). For example, when another person has a happy expression, we may infer that things are going well and thus behaviour is unaltered. However, a sad expression may suggest that help is needed, causing behaviour to change accordingly (Van Kleef, 2009). Recent research has also suggested that emotional expressions within the lunar survival task (Hall & Weston, 1970) may hamper persuasion owing to a variety of inferences made about the purpose of the emotional expression (Wang, Lucas, Khooshabeh, de Melo & Gratch, 2015).

The Lunar Survival Task involves participants ranking items in order of importance for survival following a crashed expedition. They are then asked to discuss their rankings with a virtual team member who, in response to the accuracy of their ratings (based on NASA guidance), expresses facial emotions (happy/angry) or maintains a neutral expression. Persuasion is then measured by the degree to which participants change their original rankings after the discussion. In research by Wang et al. (2015), emotional expressions reduced persuasion more when targeted at leaders and when considered inappropriate, in contrast to emotions when targeted at followers. While the hierarchy dynamics involved within the lunar task do not directly translate to the demands of the director task (in that there is no specific power imbalance between the director and the participant), these results do appear to suggest that the emotion of the person giving the instruction may impact a participant’s response. It may also be argued that there is a level of hierarchical instruction within the director task, in that the participant is following instructions from the another (the director). It may, therefore, be tentatively postulated that the director’s emotion may impact a participant’s perspective taking, influencing decisions regarding which perspectives should be used as the basis of action. As such, considering the paucity of research in this domain, the current study should be considered as a first step towards exploring whether emotion can help or hinder visual perspective taking.

It has also been suggested that there is a link between perspective taking and contagious emotional affect upon viewing faces, whereby those who were asked to take the perspective of another person show spontaneous evidence of emotional contagion, mirroring the affect displayed (Lamm, Porges, Cacioppo, & Decety, 2008). Here, it may be asserted that in trying to understand the target person’s situation, participants might have engaged in perspective taking and thus shared the target person's emotion. In other words, emotional contagion may be a by-product of perspective taking. However, it has also been suggested that such emotional contagion may be an innate propensity with evolutionary basis, allowing us to better understand and interact with others (Nesse, 1990). Indeed, research has suggested that listening to a speech with an emotional voice can induce congruent mood states in the listener but that this effect is independent of perspective taking, as it appeared that participants did not always have to see a situation from the perspective of another to share an emotional response (Neumann & Strack, 2000). There is, therefore, an unclear picture as to whether perspective taking is aided by emotional displays. The current research will explore whether the emotional state of the director may impact perspective taking performance. We hypothesise that when there is a difference between the emotional expression and the liking information given, this may highlight the perspective conflict between the participant and director, which may aid performance.

Finally, the current research will explore whether the interaction of emotion and knowledge of liking will impact perspective taking. Specifically, building on the work of Wang et al. (2015), we will examine if ToM accuracy is affected by knowledge of whether the director’s displayed emotions are congruent or incongruent with his stated likes/dislikes. If an emotional expression is congruent with the director’s (dis)liking (i.e. they are smiling because they can see something they like, or they are frowning because they can see something they dislike) accuracy may be improved, while if the director’s emotion is incongruent with their pre-defined (dis)likes, ‘persuasion’ would may be hampered, and participants may be less likely to follow the instruction (Wang et al., 2015). Such assertions, however, require further exploration. Indeed, incongruency may also highlight the perspective difference between the participant and director, resulting in better performance (similarly to ambiguous compared to relational instructions in Apperly et al., 2010). The current research will, therefore, employ the director task to explore the interactive effect of knowledge of another’s (dis)likes and their emotion, in a manner that is more akin to the type of ToM that (mal) functions in everyday social interactions (von Scheve, 2012).

The current study, therefore, aims to advance our understanding of ToM by introducing emotion and knowledge of liking into tasks which have hitherto largely ignored such potential variables. Specifically, it examines whether emotion may impact ToM use by manipulating the facial expression of the director.^1^ The following predictions were made: (1) when the director has a pre-stated liking of the target object or a dislike for competitor object, in both cases there is congruency with the choice of the correct target object (they do not have to deal with an avoidance of liking, rather they follow his liking). It is anticipated that in such cases, a relative increase in accuracy may be observed. (2) When the director has a stated liking for competitor object or a dislike for target object, the choice of the incorrect competitor object may be more incongruent. Here it is, therefore, predicted that performance will be impaired as participants have to deal with the avoidance of another’s liking, leading to relatively decreased accuracy. (3) Relative to the director having a neutral expression (group 1), when he has a congruent facial expression (smiling in the presence of an object which he is known to like^2^ or frowning in the presence of an object he is known to dislike; group 2 is expected to enhance participant performance, as the expression is deemed congruent (Wang et al., 2015). (4) An incongruent director expression (smiling in the presence of a disliked object or frowning where the object is liked; group 3) is expected to accentuate the difference in perspectives, aiding in the integration of the director’s perspective with his instruction and thus increasing accuracy, while reducing differences between the liking conditions.

## Method

### Participants

A total of 164 participants took part in the research (31 males (19%), *M* age = 20.65, SD age = 5.34), 58 in study 1 (10 males (17%), *M* age = 21.37, SD age = 6.03), 53 in study 2 (10 males (19%), *M* age = 19.26, SD age = 5.05) and 53 in study 3 (11 males (21%), *M* age = 19.73, SD age = 5.05). A priori power analyses suggested that with alpha set at 0.05, to detect a medium effect size of 0.25 at an observed power of 0.95, a total of 138 participants would be needed (G*Power; Faul, Erdfelder, Buchner & Lang, 2009). The study took approximately 50 min, and participants received course credits or £5 for completion. Ethical approval was granted by the Departmental Research Ethics Committee.

### Design

The within-subject variables were object (target × competitor) and avatar knowledge (likes × dislikes). The between-subject factor was avatar emotion (happy, sad or neutral). As such there were three groups of participants: For group 1, the avatar’s facial emotion was neutral, for group 2 the facial emotion was congruent with the avatar’s stated (dis)like of the object (i.e. if the Director liked the target or competitor object, his facial expression was positive; if he disliked the object, his facial expression was negative), and for group 3 the facial emotion was incongruent (i.e. if the Director liked the target or competitor object, his facial expression was negative; if he disliked the object, his facial expression was positive).^3^ Accuracy rates and reaction times were compared for correct object selection for the critical instruction.

Facial emotion was a between-subjects factor to limit the number of trials for participants, and to also avoid any potential practice and/or learning effects. Keeping the facial emotion constant per group also allowed for any specific effects of congruency/incongruency to be analysed, with mixing emotional congruency within a single task potentially masking this. To account for differences between the groups, empathy was measured using the Emotional Quotient (Baron-Cohen & Wheelwright, 2004) as this may have affected task performance.

### Materials

The stimuli were modified from those used by Apperly et al. (2010). Participants were presented with a 4 × 4 static grid array with eight objects present. Five slots were occluded from the view of the director (male figure with a male voice), with the remaining slots visible to the participant and the director. There were four patterns of occluded slots, and the overall image was sized at 720 × 540 pixels. Participants were shown an example grid array from the perspective of the director and themselves, making it clear objects in the occluded slots were not mutually visible, and were also informed that they would be told of the director’s like or disliking for particular item(s) in the grid prior to each trial.

Verbal instructions, using pre-recorded.wav files, were given by the director to “Move the [noun] left/right/up/down one slot” and each lasted approximately 800 ms. There were between 3 and 5 instructions per grid array, with a total of 128 instructions across 32 grid arrays. In the 16 experimental grids, the critical instruction could refer to both a mutually visible target object and a competitor object that was only visible to the participant. In 16 matched control grids, the competitor object was replaced by a filler item unrelated to the referent in the critical instruction. Specifically, the referent was the superordinate category which included both the target and distracter objects, but not the filler object. All other instructions were fillers referring to mutually visible objects (see Table 1 for example of items and instructions).

**Table 1:** Example items and instructions

Target	Competitor	Filler	Instruction referent
Red apple	Green apple	Watch	Apple
Tie	Bow-tie	Screwdriver	Tie
Mouse	Computer mouse	Can-opener	Mouse
Champagne	Spectacles	Boat	Glasses
Football	Rugby ball	Card	Ball

**Fig. 1:**
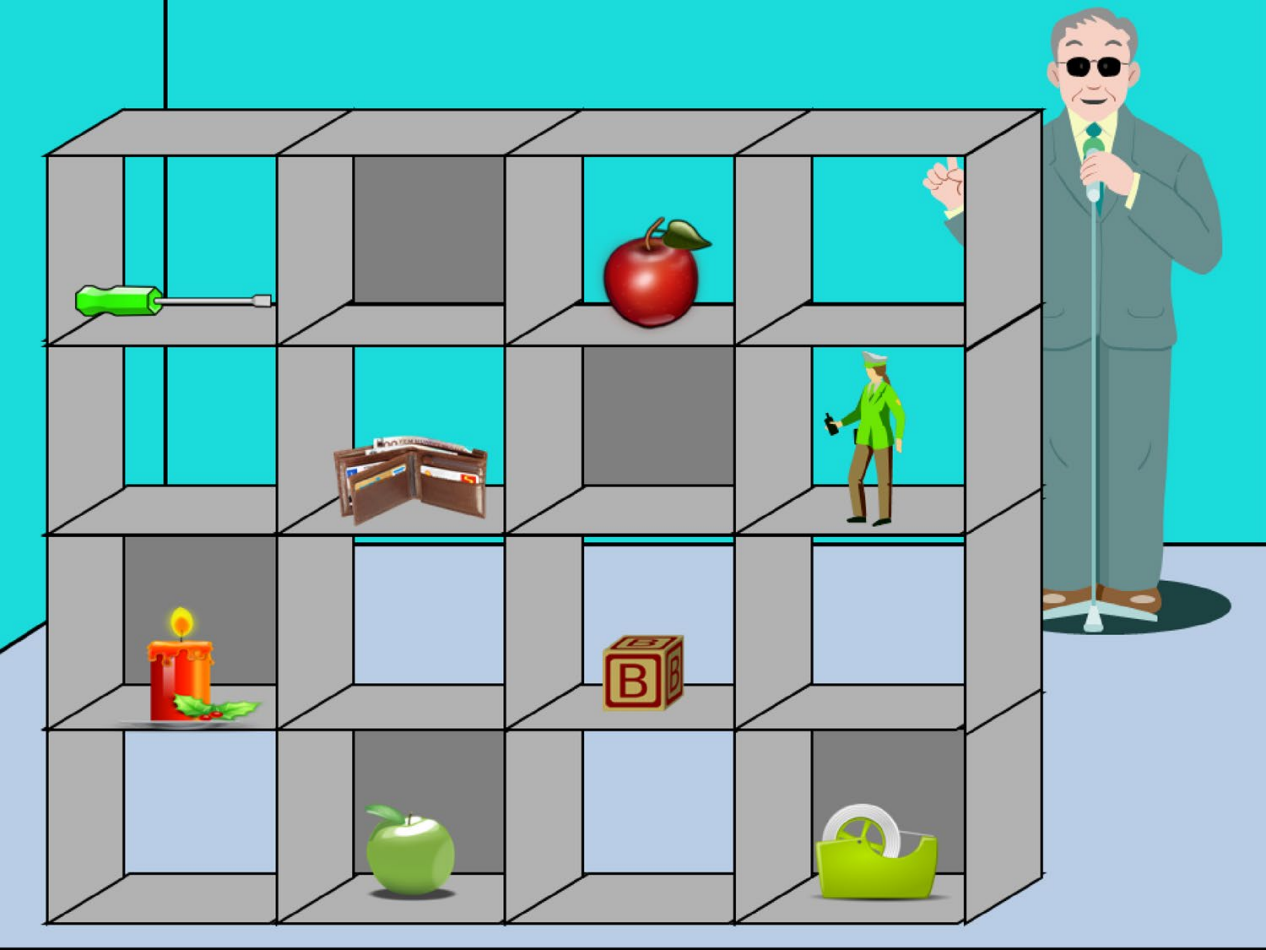
Example experimental grid for group 1: Director facial emotion = neutral, avatar knowledge = likes green apples, condition = likes competitor

**Fig. 2:**
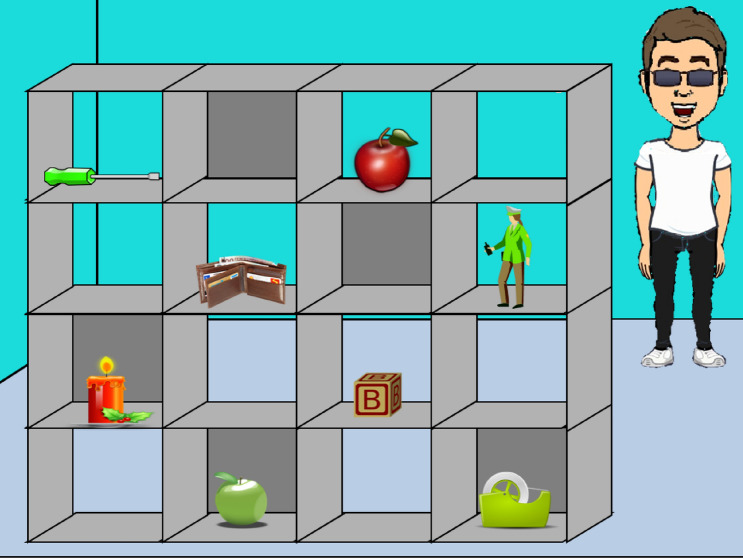
Example experimental grid for group 2: Director facial emotion = congruent, avatar knowledge = likes green apples, condition = likes competitor

**Fig. 3:**
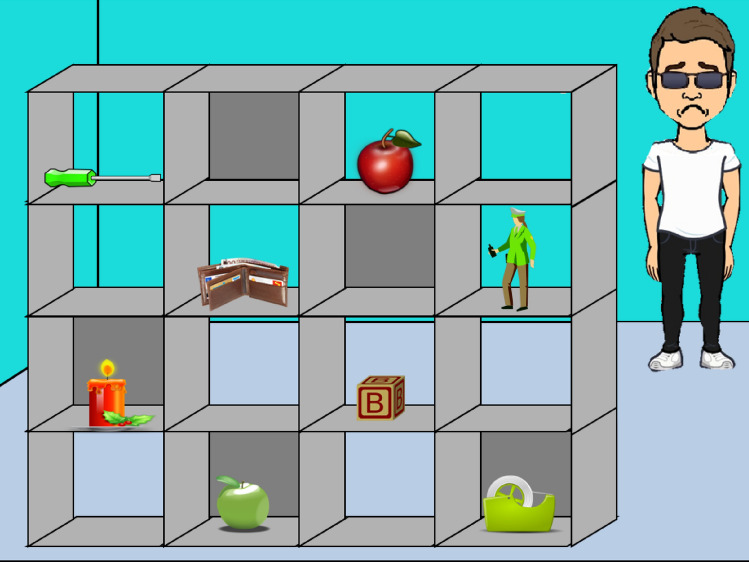
Example experimental grid for group 3: Director facial emotion = incongruent, avatar knowledge = likes green apples, condition = likes competitor

The number of instructions per grid varied between three and five, and critical instructions could occur at any position. Prior to every trial, participants were informed of the likes or dislikes of the director, which were specific to the potential referents of the following instructions. For example, the participant would be told that the director liked cats, and the instruction would be to move the animal, where the mutually visible object was a cat and the object only known/visible to the participant (i.e. in privileged ground) was a dog (like target condition). Conditions were balanced across the grids and the order was randomised. Figure 1 shows an example experimental grid where the director likes green apples and his expression is neutral (group 1, likes competitor condition). Figure 2 shows the same experimental grid and condition, with the facial emotion congruent with the avatar’s stated (dis)liking for the object (group 2). Figure 3 again shows the same experimental grid and condition, with the facial emotion incongruent with his (dis)liking (group 3). This facial emotion remained constant throughout each grid.^4^

#### Procedure

Participants were briefed and then completed the informed consent form. They then completed the Empathy Quotient scale (Baron-Cohen & Wheelwright, 2004) and were randomly allocated into one of the three facial emotion groups. They were then shown an example grid array from both their own and the director’s perspective. They were instructed to respond as quickly and accurately as possible by clicking on the object to be moved using the mouse cursor on-screen (this did not move the object on the screen, and they were reminded they did not need to recall where objects had been moved to, but to always move the object from where it was present on the screen). The experiment started with two practice grids (with no explicit feedback), followed by 32 grids for the main experiment. Each grid was preceded by a screen showing participants the director’s likes or dislikes for 2000 ms (e.g. ‘Likes cake’). The grid was then presented for 5000 ms, prior to the initial instruction. Instructions were then given at 5000-ms intervals.

## Results

Scores on the Empathy Quotient did not differ between the director emotion groups (*F* (1, 130) = 2.08, *p* = 0.13, *η*_*p*_^2^ = 0.04; neutral = 44.07 (SD = 13.51), congruent = 49.44, (SD = 11.47), incongruent = 44.90 (SD = 10.31), overall *M* = 45.96, SD = 12.23). This was, therefore, not analysed further. Internal reliability for the Empathy Quotient was also satisfactory (neutral = 0.92, congruent = 0.87, incongruent = 0.85, overall = 0.89). The gender mix, while predominantly female, also did not differ between groups (*Χ*^2^ (2) = 0.18, *p* = 0.91), and neither did age (*F* (1, 106) = 2.36, *p* = 0.10, *η*_*p*_^2^ = 0.04).

**Fig. 4:**
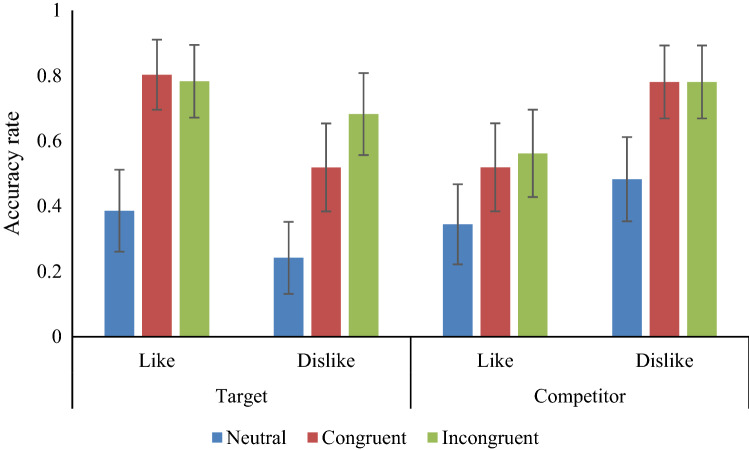
Raw accuracy rates by avatar knowledge (likes × dislikes), object (target × competitor) and avatar emotion (neutral × happy × sad). Bars = confidence intervals

Analyses were conducted using STATA 11.2. Factors of avatar knowledge (likes × dislikes), object (competitor × target) and avatar’s emotion group (neutral × congruent × incongruent) were entered into a logistic regression for accuracy (0, 1) and in a mixed linear regression for response times (accurate trials only), using xtmelogit and xtmixed commands, respectively. To control for variation in performance across items and subject, both were included as (crossed independent) random factors in both analyses, reducing the possibility of an inflation of the rate of false positives that could occur if they were treated as fixed (raw, trial-level data are available in Supplementary materials). Object and avatar knowledge were also included as random slopes within the subject random factor (Barr, 2013; Barr, Levy, Scheepers & Tily, 2013) Analyses without these random factors are presented in Appendix 1. Raw accuracy data are shown in Fig. 4.

### Accuracy (logistic regression)

There was a main effect of avatar knowledge [odds ratio (OR) = 0.14, *p* < 0.01], with a lower chance of accuracy for like than dislike, and a main effect of object with a lower chance of accuracy when the director knowledge was about the target (OR = 0.14, *p* < 0.01). There was also a main effect of director emotion, with greater chances of accuracy for both the congruent (OR = 21.04, *p* < 0.001) and incongruent (OR = 21.93, *p* < 0.001) emotion groups relative to the neutral group. There were interactions between avatar knowledge and object (*p* < 0.001) and between object and director emotion (*p* < 0.05). Full results can be seen in Table 2.

**Table 2:** Mixed effects logistic regression on accuracy for avatar knowledge × object × director emotion group (Wald *Χ*^2^ (11) = 56.11, *p* < 0.001)

	Odds ratio	*p*	95% confidence interval	
Reference: dislike				
Like	0.14	< 0.01	0.05	0.36
Reference: competitor				
Target	0.14	< 0.01	0.06	0.34
Avatar knowledge × object				
Reference: dislike/competitor				
Like target	27.91	< 0.001	7.72	100.81
Reference: neutral emotion				
Congruent emotion	21.04	< 0.001	4.41	100.36
Incongruent emotion	21.93	< 0.001	4.54	100.01
Avatar knowledge × director emotion				
Reference: dislike/neutral emotion				
Like/congruent emotion	1.12	0.86	0.40	3.15
Like/incongruent emotion	1.64	0.35	0.59	4.56
Object × director emotion				
Reference: competitor/neutral emotion				
Target/congruent emotion	1.09	0.86	0.40	2.97
Target/incongruent emotion	3.77	< 0.05	1.34	10.61
Avatar knowledge × object × director emotion				
Reference: dislike/competitor/neutral emotion				
Like/target/congruent emotion	2.43	0.22	0.60	9.96
Like/target/incongruent emotion	0.37	0.15	0.09	1.44

Random-effects parameter	Estimate (SE)	95% confidence interval	
Subject			
Standard deviation (avatar knowledge)	0.49 (0.30)	0.15	1.62
Standard deviation (object)	0.58 (0.27)	0.23	1.47
Standard deviation (constant)	3.10 (0.42)	2.37	4.05
Standard deviation (constant)	0.97 (0.46)	0.38	2.47

The likelihood ratio suggests that the random factors of subject and item account for a significant proportion of the variance in the model (*Χ*^2^ (4) = 1300.04, *p* < 0.001).

The significant two-way interactions were analysed further. For the interaction between avatar knowledge and object, the baseline was the accuracy rate for when the director disliked the competitor object (OR = 1). Relative to this, the chances of accurate responses were significantly lower when the target was disliked by the director (OR = 0.31, *p* < 0.01) and when the competitor was liked by the director (OR = 0.21, *p* < 0.001). There was no difference when the target was liked (OR = 0.86, *p* = 0.71). Full results are shown in Table 3.

**Table 3:** Avatar knowledge × object (Wald *Χ*^2^ (3) = 52.07, *p* < 0.001)

Avatar knowledge		Object	OR	*p*	95% confidence intervals	
Dislike	Competitor		1			
	Target		0.23	< 0.001	0.14	0.38
Like	Competitor		0.18	< 0.001	0.10	0.30
	Target		1.03	0.87	0.69	1.55

Random-effects parameter	Estimate (SE)	95% confidence interval	
Subject			
Standard deviation (avatar knowledge)	0.54 (0.28)	0.19	1.51
Standard deviation (object)	0.63 (0.26)	0.28	1.41
Standard deviation (constant)	3.49 (0.47)	2.69	4.54
Item			
Standard deviation (constant)	0.95 (0.46)	0.37	2.45

The likelihood ratio suggests that the random factors of subject and item account for a significant proportion of the variance in the model (*Χ*^2^ (4) = 1608.12, *p* < 0.001).

This suggests that the knowledge of the director influenced the participants accuracy, dependent on whether the object was the target or the competitor. When the target object was disliked or the competitor object liked, accuracy in selecting the correct (target) object was reduced (Table 4).

**Table 4:** Director emotion group × object (Wald *Χ*^2^ (5) = 27.80, *p* < 0.001)

Director emotion group	Object	OR	*p*	95% confidence intervals	
Neutral	Competitor	1			
	Target	0.76	0.36	0.42	1.38
Congruent	Competitor	26.54	< 0.001	5.33	132.17
	Target	30.49	< 0.001	5.97	155.80
Incongruent	Competitor	35.38	< 0.001	5.97	155.80
	Target	62.84	< 0.001	11.12	355.05

Random-effects parameter	Estimate (SE)	95% confidence interval	
Subject			
Standard deviation (avatar knowledge)	0.32 (0.49)	0.02	6.38
Standard deviation (object)	0.38 (0.44)	0.04	3.71
Standard deviation (constant)	3.28 (0.46)	2.49	4.32
Item			
Standard deviation (constant)	1.46 (0.41)	0.84	2.54

For the interaction between object and director emotion group, the baseline was the neutral emotion group, where the director knowledge was about the competitor object (OR = 1). While the accuracy of the same group when the director knowledge was about the target object was not significantly different to the baseline (OR = 0.89), the chances of accuracy were higher for all the other groups and director knowledge conditions. Specifically, for the congruent emotion group, when the director knowledge was about the competitor object (OR = 26.54) or the target object (OR = 30.49) chances of accurate responses were higher. For the incongruent emotion group, the same pattern was shown (director knowledge about competitor OR = 35.38; director knowledge about target object OR = 62.84), but to a greater extent compared to the congruent emotion group.

The likelihood ratio suggests that the random factors of subject and item account for a significant proportion of the variance in the model (*Χ*^2^ (1) = 1262.56, *p* < 0.001).

The results suggest that relative to the neutral emotion group, chances of accuracy were higher in both the congruent and incongruent emotion groups, with performance generally better when the director knowledge concerned the target object in both groups.

### Response time (accurate trials only)

There were no main effects or interactions shown (all *p*’s > 0.09), though the overall model was significant. The likelihood ratio suggests that the random factors of subject and item account for a significant proportion of the variance in the model (*Χ*^2^ (1) = 386.82, *p* < 0.001). Full results can be seen in Table 5.

**Table 5:** Multi-level mixed linear regression for avatar knowledge × object × director emotion group [Wald *Χ*^2^ (11) = 22.49, *p* < 0.05)]

	Coefficient	*p*	95% confidence interval	
Reference: dislike				
Like	44.87	0.53	− 101.65	199.39
Reference: competitor				
Target	43.12	0.52	− 89.45	175.69
Avatar knowledge × object				
Reference: dislike/competitor				
Like target	− 119.81	0.19	− 300.57	60.95
Reference: neutral emotion				
Congruent emotion	51.41	0.62	− 149.79	252.61
Incongruent emotion	72.98	0.48	− 128.22	274.17
Avatar knowledge × director emotion				
Reference: dislike/neutral emotion				
Like/congruent emotion	96.37	0.37	− 114.88	307.61
Like/incongruent emotion	119.68	0.27	− 91.56	330.93
Object × director emotion				
Reference: competitor/neutral emotion				
Target/congruent emotion	158.84	0.12	− 42.28	359.96
Target/incongruent emotion	120.51	0.24	− 80.61	321.63
Avatar knowledge × object × director emotion				
Reference: dislike/competitor/neutral emotion				
Like/target/congruent emotion	− 177.42	0.21	− 457.20	102.36
Like/target/incongruent emotion	− 124.20	0.38	− 403.98	155.59
Constant	2698.67	< 0.001	2556.48	2840.85

Random-effects parameter	Estimate (SE)	95% confidence interval	
Subject			
Standard deviation (avatar knowledge)	1.30 e^−06^ (2.82 e^−06^)	1.85 e^−08^	0.00
Standard deviation (object)	100.64 (73.36)	24.11	420.03
Standard deviation (constant)	365.09 (26.85)	316.08	421.70
Item			
Standard deviation (constant)	384.14 (66.94)	273.00	540.52
Standard deviation (residual)	677.24 (40.21)	602.83	760.83

## Discussion

There is a growing awareness that the perspective taker’s ability/state may impact ToM, particularly their ability to take the visual perspective of another (e.g. Bukowski & Samson, 2016). However, it is less clear how the attributes of the target (i.e. the characteristics of the person whose perspective a participant aims to adopt) influence this visual perspective taking. Further, while emotion and (dis)like are key to real-life interactions, there has been sparse research examining how knowledge of such facets may impact visual perspective taking abilities. The current research, therefore, aimed to advance our understanding of real-world VPT (as one of the facets of ToM) by modifying factors within the director task which have hitherto largely been unchanged. Results showed that knowledge of the director’s liking/disliking appeared to impact accuracy, though this effect was magnified when in conjunction with a director displaying an emotion. Specifically, if the participants were told that the director liked the target, participant accuracy increased, while if they liked the competitor, this accuracy decreased. Conversely, when the director disliked the target, accuracy rates were lower, while when they disliked the competitor accuracy rates increased. The accuracy shown by the different director emotion groups also followed our hypotheses, with the lowest accuracy shown by the neutral emotion group, followed by the congruent and then the incongruent emotion groups, though there was little difference between the latter two.

Results did not offer support for our prediction that the incongruent facial emotion group would show reduced differences between conditions where the participant could follow the stated liking of the director (like target, dislike competitor) and where they had to avoid that liking (dislike target, like competitor). However, this group also showed generally better performance, with higher accuracy rates in the liking for competitor and dislike for target conditions, relative to the neutral emotion group. This may support Leslie and colleagues’ (2005) assertion that predicting actions based on avoidance of desire (here defined as liking) requires inhibitory control and hence is more difficult. However, the error rates in these conditions were relatively similar to the general error rates in earlier work by Apperly et al. (2010). As such, rather than observing a negative impact owing to the avoidance of avatar liking, when the director’s preference was to approach the target object (liking target, disliking competitor), we observed a facilitation in performance, with accuracy rates for these conditions higher than in previous studies (e.g. Qureshi Monk, Samson & Apperly, 2019; Apperly et al., 2010).

When the emotion expressed by the director was congruent [in that the facial expression matched the avatar’s stated (dis)liking], there was a resultant increase in accuracy, relative to when the emotion expressed was neutral, in general support of Wang et al. (2015). One interpretation of this findings may be that the congruent emotional expression accentuates the preference of the director (e.g. Berridge, 2018). Alternatively, the participant may share the director’s emotion when taking his perspective (by a process akin to emotional contagion (e.g. Lamm et al., 2008; Nesse, 1990), which may in turn heighten the impact of the preference shown (Neumann & Strack, 2000). To fully explore this, physiological measures such as facial electromyography could be incorporated into future research.

When the emotion expressed was incongruent with director’s stated (dis)like of an object, accuracy rates were also increased (relative to neutral expressions). Here, it may be postulated that incongruency accentuates the perspective difference between the participant and director (similar to the accentuation of perspective differences through video modelling; LeBlanc, Coates, Daneshvar, Charlop-Christy, Morris & Lancaster, 2003). Indeed, this saliency may have made resolving self-other interference easier, leading to an increase in accuracy rates (e.g. Keysar et al. 2003). An alternative interpretation of the current findings is that because participants in the congruent and incongruent groups saw more diverse stimuli (both happy and sad expressions) compared to the neutral group, they saw the directors to be more agentive/intentional. This may explain the enhanced accuracy in those two groups, without the effect being caused by the (mis)match of preferences and emotions. Future research using a within-subject design is however required to examine the veracity of this alternative perspective.

This research expands the literature surrounding the Director task (e.g. Barr, 2008; Apperly et al., 2010; Keysar, Lin & Barr 2003; Symeonidou, Dumontheil, Chow & Breheny 2016) by incorporating knowledge of liking and emotional expression. The current findings suggest that when knowledge of avatar liking is positively related to the target object selection, ToM is facilitated. Emotional expression, when congruent with the stated liking/disliking of the object, accentuates this effect, whereas when that emotional expression is incongruent, the perspective difference between the director and participant may become more salient, again facilitating the use of ToM. Indeed, this may be another demonstration that participants understand the director has a different perspective and different beliefs to themselves, but they have a problem in using that knowledge (Apperly et al., 2010).

It should be noted that in the current study, congruency (between the director emotion and liking) was broadly defined. Here, we refer to congruency in cases where there was a liked item on the display (either hidden or visible to the director) and the director smiled, and incongruency where a disliked object accompanied an unhappy expression. Congruency is, therefore, defined from the participants' privileged point of view, based on the information we supplied during testing (e.g. the director likes apples). Whether or not the avatar can see the object (or it is obscured) could also be manipulated and would introduce another level of complexity to the task. We, therefore, highlight this as a potential avenue for future research which would extend further our understanding of how knowledge of another’s likes/dislikes and their apparent emotion may impact ToM. Furthermore, as the emotional expression of the director remained static throughout a grid, responses to other items/instructions (other than the critical question) could have been affected, insomuch that the emotion is not relevant to them. The expression was, however, contextually relevant to the critical instruction. In addition, the length of each trial/grid was relatively short, and we were only interested in the critical instruction. As such, while the pragmatics of the experimental context may have been affected, we do not think that this will have significantly impacted the pattern of results. Nevertheless, future research may benefit from using dynamic directors whose facial emotions (and indeed lip movements) could be synced to instructions and critical items. Finally, we note that the neutral avatar (from the original Apperly et al., 2010 task) was visually dissimilar from the happy and sad agents designed for this task and while there was no discernible pattern in the data to suggest that this elicited significant differences in attentional pull, there is a slight potential for bias which cannot be ruled out.

In conclusion, the current study is the first to incorporate both emotion and knowledge of another’s likes/dislikes into the Director task, with results suggesting that knowledge (of like for target, dislike for competitor) facilitates ToM use, while emotion, whether congruent (positive emotion × like; negative emotion × dislike) or incongruent (positive emotion × dislike; negative emotion × like), also improves performance. The study is, therefore, a first step towards incorporating key aspects of day-to-day communication into typical visual perspective taking tasks.

### Electronic supplementary material

Below is the link to the electronic supplementary material.

Supplementary file1 (TXT 2393 kb)

## Appendix 1: analyses without random effects

### Accuracy (logistic regression)

There was a main effect of avatar liking (odds ratio (OR) = 0.56, *p* < 0.01), with a lower chance of accuracy for like than dislike, and a main effect of object with a lower chance of accuracy when the object referred to was the target (OR = 0.34, *p* < 0.001). There was also a main effect of direction emotion, with greater chances of accuracy for both the congruent (OR = 3.81, *p* < 0.001) and incongruent (OR = 3.81, *p* < 0.001) emotion groups relative to the neutral group. There were interactions between avatar knowledge and object (*p* < 0.001), between avatar knowledge and director emotion (*p* = 0.03) and between object and director emotion (*p* = 0.04), as well as a three-way interaction between avatar knowledge, object and director group (*p* < 0.01). Full results can be seen in Table

**Table 6:** Mixed effects logistic regression on accuracy for avatar knowledge × object × director emotion group (Likelihood ratio *Χ*^2^ (11) = 495.36, *p* < 0.001)

	Odds ratio	*p*	95% confidence interval	
Reference: dislike				
Like	0.56	< 0.01	0.38	0.84
Reference: competitor				
Target	0.34	< 0.001	0.24	0.48
Avatar knowledge × object				
Reference: dislike/competitor				
Like target	3.50	< 0.001	2.16	5.69
Reference: neutral emotion				
Congruent emotion	3.81	< 0.001	2.51	5.78
Incongruent emotion	3.81	< 0.001	2.51	5.78
Avatar knowledge × director emotion				
Reference: dislike/neutral emotion				
Like/congruent emotion	0.54	0.03	0.30	0.96
Like/incongruent emotion	0.64	0.13	0.36	1.14
Object × director emotion				
Reference: competitor/neutral emotion				
Target/congruent emotion	0.89	0.66	0.52	1.52
Target/incongruent emotion	1.77	0.04	1.02	3.06
Avatar knowledge × object × director emotion				
Reference: dislike/competitor/neutral emotion				
Like/target/congruent emotion	3.56	< 0.01	1.64	7.74
Like/target/incongruent emotion	1.33	0.47	0.61	2.90

^6^.

The three-way interaction was analysed further. For the interaction between avatar knowledge and object, the baseline was dislike competitor (OR = 1). Relative to this, accuracy chances were higher for when the competitor was disliked and target liked for the congruent and incongruent emotion groups. This was also the case for disliking the target for the incongruent emotion group. Accuracy chances were lower when disliking the target, liking the competitor and liking the target for the neutral emotion group. Full results are shown in Table

**Table 7:** Odds ratios by object, avatar knowledge and director emotion group (baseline = neutral emotional director group, dislike, competitor), (Likelihood ratio *Χ*^2^ (11) = 495.36, *p* < 0.001)

Avatar knowledge	Object	Director emotion group	OR	*p*	95% confidence intervals	
Dislike	Competitor	Neutral	1			
		Congruent	3.81	< 0.001	2.51	5.78
		Incongruent	3.81	< 0.001	2.51	5.78
	Target	Neutral	0.34	< 0.001	0.24	0.48
		Congruent	1.15	0.46	0.79	1.69
		Incongruent	2.30	< 0.001	1.55	3.42
Like	Competitor	Neutral	0.56	< 0.01	0.38	0.84
		Congruent	1.16	0.46	0.79	1.70
		Incongruent	1.37	0.11	0.93	2.02
	Target	Neutral	0.67	0.02	0.49	0.94
		Congruent	4.37	< 0.001	2.80	6.81
		Incongruent	3.86	< 0.001	2.50	5.96

^7^.

### Response time (accurate trials only)

There were no main effects or interactions shown (all *p*’s > 0.06), though the overall model was significant. Full results can be seen in Table

**Table 8:** Linear regression for avatar knowledge × object × director emotion group (*F* (11, 1616) = 3.39, *p* < 0.01, *r*^2^ = 0.02)

	Coefficient	*p*	95% confidence interval	
Reference: dislike				
Like	172.91	0.11	− 36.33	382.14
Reference: competitor				
Target	156.79	0.09	− 28.81	342.38
Avatar knowledge × object				
Reference: dislike/competitor				
Like target	− 234.67	0.08	− 498.05	28.71
Reference: neutral emotion				
Congruent emotion	65.36	0.45	− 103.32	234.04
Incongruent emotion	162.08	0.06	− 6.61	330.76
Avatar knowledge × director emotion				
Reference: dislike/neutral emotion				
Like/congruent emotion	− 45.62	0.74	− 311.24	220.01
Like/incongruent emotion	44.24	0.74	− 218.99	307.46
Object × director emotion				
Reference: competitor/neutral emotion				
Target/congruent emotion	− 79.42	0.53	− 326.25	167.41
Target/incongruent emotion	34.68	0.78	− 204.09	273.46
Avatar knowledge × object × director emotion				
Reference: dislike/competitor/neutral emotion				
Like/target/congruent emotion	35.42	0.84	− 316.48	387.33
Like/target/incongruent emotion	− 197.87	0.26	− 542.77	147.02
Constant	2744.93	< 0.001	2609.71	2880.15

^8^.

## Footnotes

1 The neutral Director was the original use in Apperly et al. (2010). Those with emotional expressions were designed for the current study.

2 NB., Here, congruency refers to whether the emotional expression of the other (the director) is in line with their stated (dis)liking of object, irrespective of whether this object can be seen or not (more information in the “Method”).

3 In other words, whether the emotion is in line with the director’s stated (dis)liking of an object, irrespective of whether this object can be seen by the avatar or it is obscured from view.

4 The neutral director was taken from Apperly et al., (2010), the directors with happy and sad emotions were piloted pre-test to ensure the validity of their emotional expression.
